# Phosphorus Magnetic Resonance Spectroscopy (^31^P MRS) and Cardiovascular Disease: The Importance of Energy

**DOI:** 10.3390/medicina59010174

**Published:** 2023-01-15

**Authors:** Vasiliki Tsampasian, Donnie Cameron, Rashed Sobhan, George Bazoukis, Vassilios S. Vassiliou

**Affiliations:** 1 Norwich Medical School, University of East Anglia, Bob Champion Research & Education Building, Research Park, Rosalind Franklin Rd, Norwich NR4 7UQ, UK; 2 C.J. Gorter MRI Center, Department of Radiology, Leiden University Medical Centre, 2333 ZA Leiden, The Netherlands; 3 Department of Cardiology, Larnaca General Hospital, Larnaca 6301, Cyprus; 4 Department of Basic and Clinical Sciences, University of Nicosia Medical School, Nicosia 2417, Cyprus

**Keywords:** phosphorus magnetic resonance spectroscopy (^31^P MRS), myocardial energetics, cardiovascular disease, cardiovascular imaging, cardiac magnetic resonance imaging

## Abstract

*Background and Objectives*: The heart is the organ with the highest metabolic demand in the body, and it relies on high ATP turnover and efficient energy substrate utilisation in order to function normally. The derangement of myocardial energetics may lead to abnormalities in cardiac metabolism, which herald the symptoms of heart failure (HF). In addition, phosphorus magnetic resonance spectroscopy (^31^P MRS) is the only available non-invasive method that allows clinicians and researchers to evaluate the myocardial metabolic state in vivo. This review summarises the importance of myocardial energetics and provides a systematic review of all the available research studies utilising ^31^P MRS to evaluate patients with a range of cardiac pathologies. *Materials and Methods*: We have performed a systematic review of all available studies that used ^31^P MRS for the investigation of myocardial energetics in cardiovascular disease. *Results*: A systematic search of the Medline database, the Cochrane library, and Web of Science yielded 1092 results, out of which 62 studies were included in the systematic review. The ^31^P MRS has been used in numerous studies and has demonstrated that impaired myocardial energetics is often the beginning of pathological processes in several cardiac pathologies. *Conclusions*: The ^31^P MRS has become a valuable tool in the understanding of myocardial metabolic changes and their impact on the diagnosis, risk stratification, and prognosis of patients with cardiovascular diseases.

## 1. Introduction

Myocardial energetics represent one of the most important biochemical processes in the human body. A heart that functions well also has a normal cardiac metabolic profile. Derangement of this metabolic profile heralds the pathophysiological processes that subsequently lead to heart failure symptoms. This “engine out of fuel,” as the failing heart has previously been described [1], needs to be diagnosed appropriately and in a timely fashion, but even more importantly, the mechanism behind this failure needs to be understood.

The correlation of biochemical pathways with metabolic function and energetic state in active tissues is a challenging project that researchers have acknowledged since the late 1970s [2]. At the time, the very first studies using 31-phosphorus magnetic resonance spectroscopy (^31^P MRS) began. These were ex vivo studies that, given the limitations of spatial localization techniques, focused on the energetics of animal hearts [2,3,4,5]. However, with the advancement of surface coils and improvements in spatial localization techniques, the first in vivo studies of human hearts emerged [6,7]. Since then, ^31^P MRS has significantly contributed to clinical research, and by providing invaluable insights on cardiac energetics, it has become a powerful non-invasive tool that enables the study of the most metabolically demanding organ in vivo, the heart.

This comprehensive review examines the link between cardiac energetics and cardiovascular disease and the important role that ^31^P MRS plays in the thorough assessment of this link. It also provides a systematic review of the available studies in the literature that have used ^31^P MRS for the assessment of cardiovascular diseases in humans and the prognostic implications of their findings.

## 2. Materials and Methods

### 2.1. Search Strategy

We have performed a systematic review of all available studies that used ^31^P MRS for the investigation of myocardial energetics in cardiovascular disease. The two independent investigators (V.T. and G.B.) performed a systematic search in the Medline database, the Cochrane library, and Web of Science from inception to 30 September 2022. No limitations were used in the search strategy. The following algorithm was used to retrieve all relevant studies for “cardiovascular disease AND (^31^P spectroscopy OR phosphorus spectroscopy)”.

### 2.2. Eligibility Criteria

We considered eligible all studies that used ^31^P MRS for the investigation of myocardial energetics in cardiovascular disease. In addition, we excluded studies that were conducted in animals or ex vivo.

### 2.3. Data Collection Process

The following data were extracted for each included study: publication data (first author, year of publication), patient characteristics (number of patients, mean age, gender, type of cardiomyopathy), and the reported outcomes.

## 3. Results

**Figure 1:**
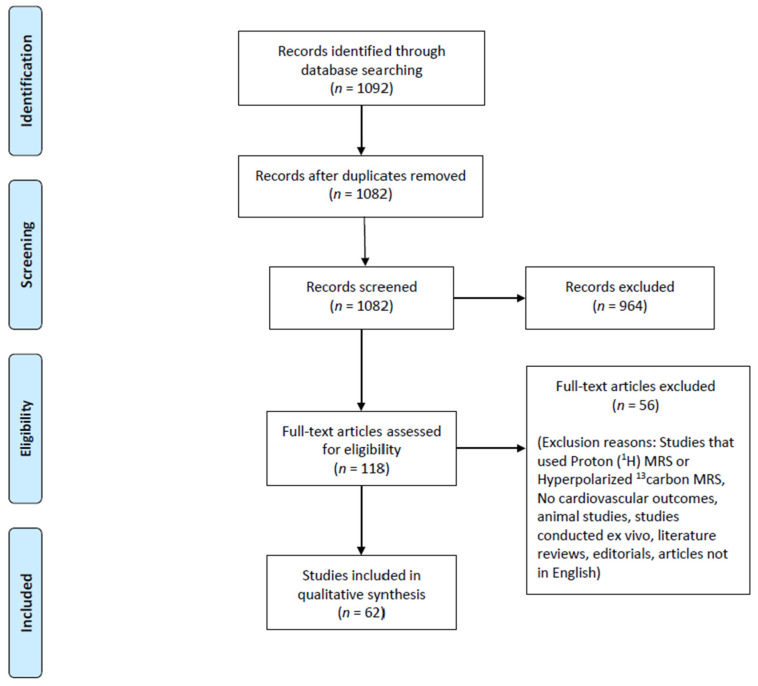
PRISMA flow diagram of the study selection process.

Our search strategy returned 1092 possible relevant studies. Of them, 10 studies were excluded as duplicate records, 964 studies were excluded at the title/abstract level, while 56 studies were excluded at the full-text level. As a result, 62 studies were finally included in the review. The Preferred Reporting Items for Systematic Reviews and Meta-analyses (PRISMA) flow diagram of the study population process is shown in Figure 1. Table 1 provides a detailed description of all the included studies, their study population cohorts, the cardiovascular diseases examined, and their major outcomes.

## 4. Discussion

### 4.1. Myocardial Energetics

The heart has been described as the organ with the highest metabolic demand, consuming 8% of the total body adenosine triphosphate (ATP) [67,68]. Considering that the heart only accounts for ~0.5% of the total body weight, it is estimated that it uses 20 to 30 times its weight in ATP to maintain its metabolic homeostasis [68,69]. The cardiac metabolic phenotype is not only highly demanding but also quite dynamic, as its function depends on the continuous production of ATP. If this copious and uninterrupted production of ATP is not maintained, then the heart would be deprived of energy within less than 10 s [67,70].

The healthy adult heart primarily uses free fatty acids and glucose as its main energy substrates [71,72,73], while the rest of the energy requirements are fulfilled by alternative energy substrates such as lactate, ketones, and amino acids [67,70]. The alterations in cardiac energy demands and availability of the substrates lead to shifts in energy substrate utilisation, a feature described as “metabolic flexibility” [70,71]. This reflects the ability of the heart to switch to different energy substrates in order to maintain its requirements [70,71].

**Figure 2:**
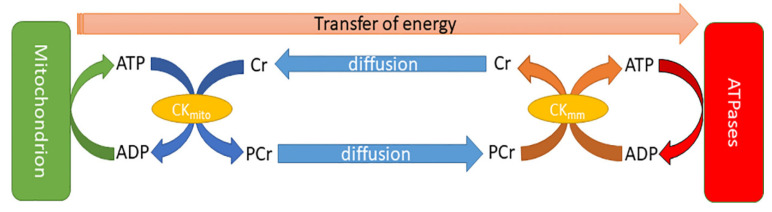
Schematic of energy transfer through the cell. Reproduced with permission from Watson et al. [75] under a Creative Commons Attribution 4.0 International License.

In order to meet these high metabolic demands, the adult heart uses the above energy substrates to generate ample amounts of ATP, mainly through mitochondrial oxidative phosphorylation, which makes up to 95% of myocardial ATP, while a smaller amount is generated through glycolysis [70,71]. The mitochondrial oxidative phosphorylation has a key role in the generation, transfer, and utilisation of ATP by the myofibrils of the cardiomyocytes. In addition, through the creatine kinase (CK) “shuttle” system, high-energy phosphate is transferred from the ATP produced in the mitochondria to creatine (Cr), generating in this way phosphocreatine (PCr) and adenosine diphosphate (ADP). The PCr, a much smaller molecule than ATP, is then rapidly diffused from the mitochondria to the sarcomeres that comprise the myofibrils. In the myofibrils, CK catalyses the formation of ATP and Cr. The ATP is then used by the ATPases and contributes to cell contraction, while Cr is diffused back into the mitochondria [1,74] (Figure 2).

The PCr is the primary energy reserve metabolite in the heart, with its concentration being twice that of ATP phosphorylation [76]. However, through the CK-mediated reaction, ATP production with the use of PCr is ~10 times faster than the rate of ATP production by oxidative phosphorylation [77]. In situations of high metabolic demand, when the rate of ATP use exceeds that of its production, the PCr/CK system therefore works as a buffer to maintain homeostasis, as the use of PCr via the CK reaction/catalysis helps in maintaining ATP at a stable normal level of phosphorylation [1,76]. Indeed, in heart failure—regardless of the aetiology—the PCr level falls in order to help maintain the ATP at a normal level [1]. While this may seem like an appropriate compensatory mechanism, it is certainly not sufficient to maintain the high metabolic demand of the cardiomyocytes. In a disease state, the failing heart is much less energy efficient, as mitochondrial dysfunction and shifts in energy substrate utilisation lead to a significant reduction in ATP production [73,78]. Additionally, PCr has been shown to decrease in pathological cardiac hypertrophy and failure as a result of the significant ATP supply-demand mismatch and partly due to a loss of creatine levels [76]. This is followed by a progressive ATP decrease [74,77]. It is therefore clear that a change in the PCr or ATP levels, or the ratio PCr/ATP, signifies a metabolic derangement that proclaims the failure of the heart. In the failing myocardium, the decrease in creatine levels occurs earlier and is faster than the reduction in ATP levels [77,79,80]. The ATP level is ~30% lower than in the normal myocardium, with the respective reduction of creatine levels being up to 50–70% in severely failing myocardium [77,80]. As creatine, PCr, and ATP levels all reduce significantly, it is likely that the PCr/ATP ratio underestimates the severity of the PCr decrease [77,79]. In addition, although previous studies have shown that the PCr/ATP ratio in the healthy myocardium is maintained, there is evidence to suggest that stress may lead to its reduction even in the absence of any underlying cardiac pathology [33,81].

### 4.2. Assessment of Myocardial Energetics with ^31^P MRS

The ^31^P MRS is a non-invasive technique that provides unique insights into the abovementioned intracellular parameters through the detection of phosphorus-containing metabolites. It therefore allows an accurate evaluation of the myocardial energetic state through measurement of the myocardial PCr/ATP ratio as well as absolute levels of high-energy phosphates [82,83]. On most magnetic resonance imaging (MRI) scanners, specialised scanner software and additional hardware, including a broadband amplifier, receive system, and radiofrequency (RF) coils, are needed to permit the acquisition of ^31^P MRS data [84]. In addition, for optimised data collection and correct metabolite quantification, the RF excitation pulse bandwidth must be large enough to excite all the relevant metabolites homogeneously [84].

The excitation of ^31^P nuclei with RF signals is required for ^31^P MRS. However, in following the excitation of the nuclei, the induced signal is recorded in the receiver coils of the scanner after the RF energy is switched off [85]. The amplitude of the received signal reflects the number of nuclei present in the interrogated tissue [85]. The resonance frequency of the nuclei being interrogated depends on their chemical environment; a phenomenon described as ‘chemical shift’ [75,85]. This is a result of different nuclei being surrounded by a different number and unique spatial location of the surrounding electrons (i.e., different shielding environments) [75,85]. Further, although the resonance frequency depends on the strength of the magnetic field, the chemical shift, as quantified in parts per million (ppm), does not alter.

**Figure 3:**
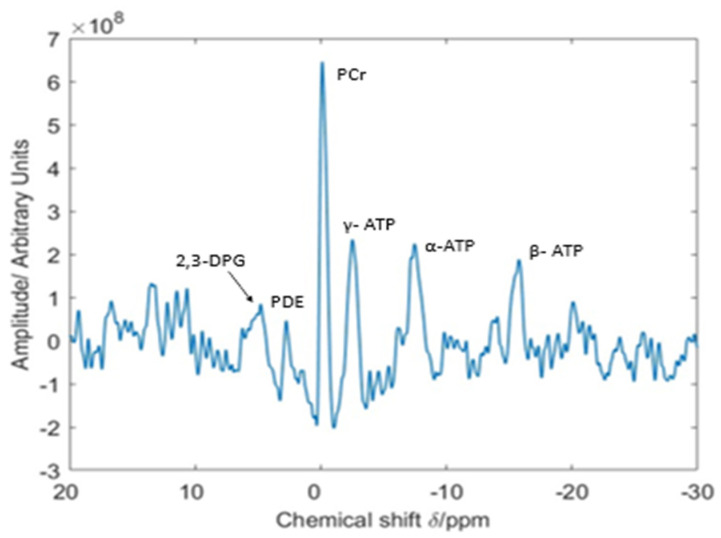
Example of a typical ^31^P cardiac spectrum demonstrating peaks for PCr, 2,3-DPG, PDE, and the three phosphorus nuclei of ATP (γ-ATP, α-ATP, and β-ATP). Image courtesy of the University of East Anglia. 2,3-DPG, 2,3-Diphosphoglycerate; PDE, phosphodiesters; PCr, phosphocreatine; ATP, adenosine triphosphate.

The typical ^31^P cardiac spectrum demonstrates peaks for phosphocreatine and the three phosphorus nuclei of ATP (γ-ATP, α-ATP, and β-ATP). The area under the peak is proportional to the relative concentrations of these metabolites [1] (Figure 3).

**Figure 4:**
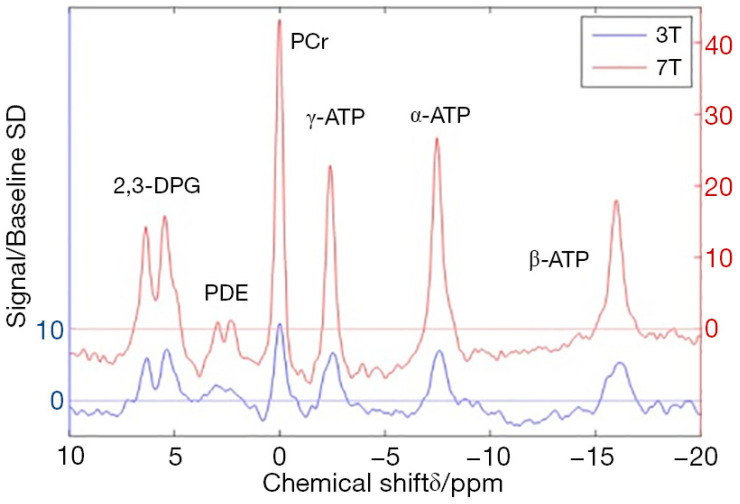
Comparison of cardiac ^31^Phosphorus spectra acquired from the same individual at 3 and 7 Tesla field strengths real part of the spectrum from a voxel in the middle of the interventricular septum. The spectra were apodized with an exponential filter (matched to the fitted PCr linewidth), first order phase corrected, and normalized to the resulting baseline noise standard deviation. Higher magnetic field yields higher signal-to-noise ratio (SNR). The SNR is markedly higher for the 7T data. Note that the y-axes are offset for clarity. Reproduced with permission from Rodgers et al. [87] under a Creative Commons Attribution 4.0 International License. SD: standard deviation; 2,3-DPG, 2,3-Diphosphoglycerate; PDE, phosphodiesters; PCr, phosphocreatine; ATP, adenosine triphosphate; ppm, parts per million.

The cardiac ^31^P MRS is typically performed on MR scanners with a field strength of 1.5 or 3 Tesla(T), although studies using a higher field strength of 7T have been reported [86,87]. As the field strength increases, the frequencies, as reflected by the spectral peaks, are more separated and therefore better quantified [75,87] (Figure 4).

A cardiac ^31^P spectrum can be obtained using a variety of techniques that help in the identification of signals from a region of interest. In brief, these include the following: (a) Depth-resolved surface coil spectroscopy (DRESS): a single slice parallel to the coil is selectively excited by an MRI gradient. (b) Image selected in-vivo spectroscopy (ISIS): data are acquired using data from inversion pulses from consecutive cycles, and voxel localisation is achieved using a slice-based intersection strategy. (c) One-, two- and three- dimensional (1D, 2D, and 3D) chemical shift imaging (CSI): this technique uses phase encoding for spatial localisation, and the spectra can be acquired in a column of voxels (1D), a plane (2D), or a block (3D) of voxels [75,88,89]. The CSI has seen more widespread use in recent years, and is often applied with saturation bands to minimise ^31^P contributions from skeletal muscle and the liver [75,89].

Using one or a combination of the aforementioned techniques, a localised cardiac ^31^P spectrum can be acquired. However, through analysis of this spectrum, the PCr/ATP ratio can be determined, allowing in this way an evaluation of the cardiac energetic state. In addition, the cellular pH (from the chemical shift of inorganic phosphate (Pi) relative to PCr) can be indirectly deduced from the acquired spectra [20,85,90]. Apart from absolute measurements, cardiac ^31^P MRS can also provide a unique insight into dynamic changes in the cellular metabolism through assessment of the CK flux and the rate of ATP generation [18,24,30]. Furthermore, by the measurement of the CK flux, the pseudo–first-order unidirectional rate constant (*k*_f_) of CK in the ATP-generating (forward) direction is first measured. This is then multiplied by the PCr/ATP ratio to give the forward CK flux, i.e., the ATP delivery rate [24,57].

The major limitation of ^31^P MRS is its intrinsically low SNR (approximately 10^5^-fold lower than ^1^H-MRS), which is typically compensated via coarser spatial and temporal resolutions [85,87,89]. This is primarily because of the low concentrations of the metabolites being studied compared to water [85,87,89]. This drawback is reflected in longer acquisition times that may be up to approximately 30 min [87,89]. Nevertheless, the disadvantage of the low SNR can be mitigated with the use of higher stronger magnetic fields, such as 7T, if available [31,87].

### 4.3. The Role of ^31^P MRS in the Evaluation of Cardiovascular Diseases

Numerous studies have shown that, in a cardiovascular disease state, there is significant impairment of energy production mediated by mitochondrial dysfunction, alterations in energy substrate utilisation, and impaired ATP transfer and utilisation [1,68]. The ^31^P MRS is the only technique available with the ability to study this pathological alteration in cellular metabolism in vivo, which is reflected in changes in cellular ATP and PCr concentrations, the PCr/ATP ratio, as well as the rate of ATP production as reflected by the CK flux. The research community has provided promising evidence that the role of ^31^P MRS is crucial in the early detection of pathological changes of the myocardial energetics through an exponential growth of clinical research studies over the last four decades.

#### 4.3.1. Myocardial Infarction (MI) and Ischaemic Cardiomyopathy (ICM)

The cardiac energy metabolism appears to be significantly impaired both in the infarcted and in the ischaemic myocardium. Following myocardial infarction, the concentrations of both PCr and ATP metabolites fall significantly [13,18]. In addition, the CK ATP supply, reflected by the CK flux, is also significantly reduced, which is likely attributable to myocyte loss [18]. The PCr/ATP ratio is very much dependent on the presence or absence of myocardial ischaemia and heart failure secondary to ischaemic cardiomyopathy. In a study that included 27 patients with severe left anterior descending artery (LAD) disease, Yabe et al. found that the PCr/ATP ratio was significantly reduced in those with reversible ischaemia compared to those with fixed defects or healthy volunteers [12]. Weiss et al. also demonstrated that the PCr/ATP ratio significantly decreased during isometric hand-grip exercise in patients with significant coronary artery disease [8]. Interestingly, the PCr/ATP ratio was not reduced in the patients that underwent successful revascularisation, suggesting the normalisation of the metabolic parameters after a timely successful clinical intervention and normalisation of the blood supply [8]. This is in keeping with another study in which 15 patients underwent ^31^P MRS 3 weeks post MI and the PCr/ATP ratio was found to be normal in viable myocardium [15].

There is only one study that investigated the potential changes in myocardial energetics in patients with normal epicardial coronary arteries. Buchthal et al. recruited 35 female patients who had been admitted to the hospital with cardiac chest pain and had normal invasive coronary angiograms. A total of Seven of the 35 women (20%) had significantly reduced cardiac PCr/ATP ratios during handgrip exercise, suggesting potentially significant abnormal myocardial metabolism in this population [16].

#### 4.3.2. Dilated Cardiomyopathy (DCM)

The DCM exhibits impaired myocardial energetics, with reduced PCr and ATP concentrations, and PCr/ATP ratios [10,21,22,23,25,26,33]. The PCr/ATP ratio has attracted a lot of research interest in this population. However, with both the PCr and ATP metabolites being significantly reduced simultaneously, it has been noted that a seemingly mild reduction in the PCr/ATP ratio may underestimate the true impairment of myocardial metabolism [23]. Despite this weakness, it has been shown to have potentially strong implications for risk stratification and prognosis. More specifically, there is evidence suggesting that the PCr/ATP ratio has a strong correlation with the clinical severity of heart failure, as estimated by the New York Heart Association (NYHA) class, as well as the left ventricular ejection fraction (LVEF) [21,23,25]. In a study that included 39 patients with DCM, Neubauer et al. demonstrated that the PCr/ATP ratio is a significant predictor of cardiovascular mortality [53]. The CK flux is also not only markedly reduced in DCM, but it also carries important prognostic implications. In a study that included 58 patients, Bottomley et al. found that reduced myocardial CK flux was a significant predictor of all-cause mortality and HF outcomes, even after correction for NYHA class, LVEF, and race [27]. 

#### 4.3.3. Hypertrophic Cardiomyopathy (HCM)

**Figure 5:**
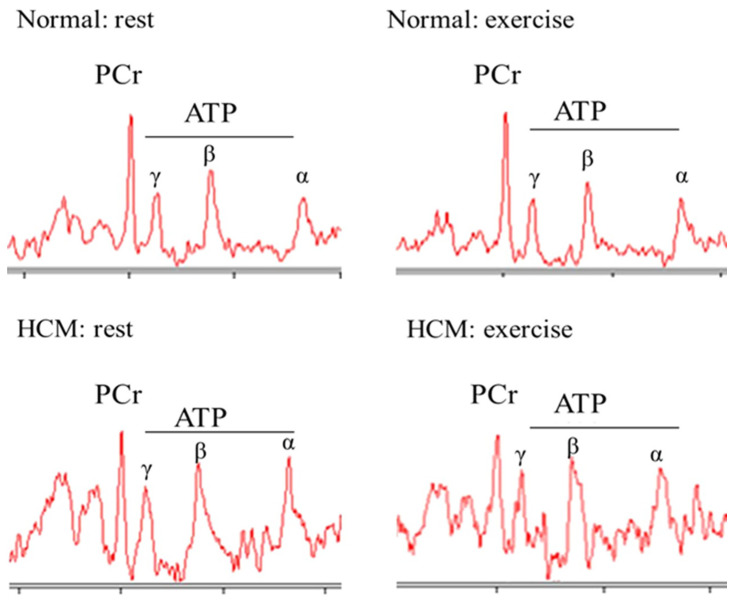
Examples of spectra in normal and hypertrophic cardiomyopathy. Reproduced with permission from Dass et al. [42] under a Creative Commons Attribution 4.0 International License.

The PCr/ATP ratio is significantly reduced in patients with HCM, highlighting the abnormal myocardial metabolic changes in this population too. Early studies from almost three decades ago demonstrated unanimously that HCM is associated with impaired myocardial energetics [20,34,37,90]. Consequently, following those early studies, research focused on specific mutations associated with the disease. In a study that included 31 patients with HCM positive for 3 mutations (beta-myosin heavy chain, cardiac troponin T, and myosin-binding protein C), Grilley et al. found that the PCr/ATP ratio was significantly reduced, and the reduction was of similar magnitude in all three disease-gene groups [38]. Another study that included 9 patients with a point mutation (Arg403Gln) in the beta-myosin heavy chain gene found significantly reduced PCr concentrations and CK flux while the PCr/ATP ratio showed a trend towards significance [41]. Furthermore, during exercise there is a pathological reduction of the PCr/ATP ratio in patients with HCM, a finding that may explain the exercise-related diastolic dysfunction in these patients [42] (Figure 5). In addition to the above, the PCr/ATP ratio has been shown to have a significant correlation with other imaging parameters derived from cardiac magnetic resonance (CMR) imaging, including T1 values and late gadolinium enhancement [28,40].

#### 4.3.4. Heart Failure with Preserved Ejection Fraction (HFpEF), Hypertensive Heart Disease (HHD), Diabetic Cardiomyopathy, and Obesity

Despite the complex and heterogeneous cohort of patients that comprise the clinical entity of HFpEF, the myocardial energetics display a uniform impairment, reflected by a significant reduction in the PCr/ATP ratio [47,50]. In addition, there is evidence suggesting that this ratio is significantly correlated with the log N-terminal pro b-type natriuretic peptide (NT-proBNP), the echocardiographic e/E’, the NYHA class, and the exercise induced pulmonary congestion that occurs in these patients [50]. Significantly reduced PCr/ATP ratios are also present in diabetic cardiomyopathy [48,51] and in hypertensive heart disease associated with systolic dysfunction [26,45]. Similarly, obesity is associated with impairment of myocardial energetics with a reduced PCr/ATP ratio at rest and failure of CK flux to increase during increased workload [49]. Weight loss appears to ameliorate the dysfunction of the cellular metabolism as it leads to an increase in the PCr/ATP ratio as an well as increase in CK flux during increased workload [49].

#### 4.3.5. Valvular Cardiomyopathy

Both aortic and mitral valve disease have been shown to have a significant impact on the myocardium [91]. This is depicted in the cardiac metabolic profile of these patients as well. More specifically, PCr/ATP is significantly decreased in symptomatic patients with aortic valve disease, and it improves post aortic valve replacement [23,52,53,55,56]. Notably, in a study that included 65 patients with aortic stenosis, Peterzan et al. found that the PCr/ATP ratio and CK flux were significantly reduced both in patients with moderate aortic stenosis, as well as those with severe aortic stenosis and normal left ventricular ejection fraction, suggesting in this way that pressure loading conditions are associated with deranged myocardial energetics even before the disease progresses to the severe stage or the presentation of symptoms [57]. In patients with mitral regurgitation, myocardial energetics are significantly impaired in symptomatic patients and in those with severe disease, while the PCr/ATP ratio is correlated with left ventricular dilatation [54].

#### 4.3.6. Heart Transplantation

The ^31^P MRS has also revealed valuable insights in the pathophysiology of cardiac allografts and is a promising non-invasive tool that can efficiently assess cardiac allograft vasculopathy (CAV). In a study that included 25 heart transplant recipients, Evanochko et al. used a ^31^P MRS stress test and found that certain patients have abnormal cardiac energetics after transplantation, as reflected by a significant change in the PCr/ATP ratio [58]. This appeared to be unrelated to the timing of the transplantation and was present in patients with normal coronary arteries as well. The authors highlight that the ^31^P MRS stress test may have an important role in the diagnosis of CAV as it is a much more sensitive means of assessing the microvasculature [58]. Similarly, Caus et al. suggest that ^31^P MRS is a promising tool able to detect impairment of myocardial energetics related to CAV, with a PCr/ATP value of 1.59 being their proposed optimal cut-off value to predict CAV with specificity and sensitivity of 100% and 72%, respectively [59]. It has to be noted, however, that ^31^P MRS is not standard as yet; hence, such cut-offs only apply to the particular methodology and the specific MRI scanner used in this study.

#### 4.3.7. Cardiovascular Research and Future Directions

As noted above, the metabolism has a central role in the pathogenesis of a range of cardiovascular diseases. As such, medical therapies targeted at the metabolic pathways that have a key role in these diseases are extremely beneficial for the patients. This has been shown for several medications that have been proven to be not only of symptomatic but also of prognostic benefit. For example, beta blockers have been shown to increase the PCr/ATP ratio by 33% in patients with heart failure after only 3 months of treatment [63], and, similarly, perhexiline has also been shown to improve the PCr/ATP ratio by 30% and the left ventricular systolic function [64]. More recently, sodium-glucose co-transporter-2 inhibitors (SGLT2i) have shown an impact on mortality and morbidity in patients with heart failure [92]. As research focuses on their mechanism of action, there is evidence suggesting that they alter myocardial energetics and improve cellular metabolism [93], as recent evidence reveals their positive impact on the PCr/ATP ratio [51].

The cardiovascular research has now shifted to treatments that focus on the improvement of myocardial energetics and metabolism, which play a pivotal role in the pathogenesis of several cardiovascular diseases. The ^31^P MRS will continue to have an instrumental part in this journey as it provides a non-invasive and accurate evaluation of the cardiac metabolic profile that helps researchers gain important insights into the complex pathophysiology of cardiac disorders. The current limiting factors in the widespread use of ^31^P MRS include the requirement of both expensive hardware and clinical expertise. Nevertheless, as research emphasises the importance of myocardial energy reserve in the pathophysiology of disease and pharmacotherapy, these issues will gradually recede.

## 5. Conclusions

The myocardial energetic compromise has proved to be an important feature in the pathophysiological process of several conditions. The ^31^P MRS is the only available non-invasive technique with the capacity to quantify metabolism in vivo, and it is, therefore, an excellent tool in the evaluation of the myocardial metabolic profile of patients with cardiac pathologies. Future studies assessing myocardial energetic phenotypes and how these are associated with clinical outcomes and prognosis will help further in understanding the impact of altered metabolism in clinical practice.

## Figures and Tables

**Table 1:** Table of studies included in the systematic review and their characteristics.

Study	Year	Participants	Major Findings
***Myocardial Infarction (MI) and Ischaemic Cardiomyopathy (ICM)***			
Weiss et al. [8]	1990	11 healthy controls 16 patients with CAD, of which 5 patients were re-examined after successful revascularisation 9 patients with non-ischaemic heart disease	^31^P NMR spectra recorded from the anterior myocardium before, during and after isometric hand-grip exercise In CAD: PCr/ATP ratio significantly decreased during exercise In non-ischaemic heart disease and in patients after revascularisation: No changes in PCr/ATP
Hardy et al. [9]	1991	12 healthy controls 9 patients with ICM 11 patients with NICM	PCr/ATP significantly lower in ICM and NICM
Neubauer et al. [10]	1992	19 healthy controls 14 patients with CAD 19 patients with DCM	In CAD: no change in PCr/ATP or PDE/ATP ratio In DCM: PCr/ATP ratio in patients with DCM tended to be lower but not significantly decreased. Marked reduction of PCr/ATP in advanced stages of heart failure. PCr/ATP ratios showed a highly significant correlation with the NYHA class
Mitsunami et al. [11]	1992	9 healthy controls 12 patients with old MI	PCr and ATP were significantly reduced in patients with old MI compared to control patients.
Yabe et al. [12]	1994	27 patients with CAD (severe LAD) 11 healthy controls	PCr/ATP ratio significantly lower in all CAD patients (lower in those with fixed thallium defects compared to those with reversible ischaemia) Contrary to normal subjects or patients with fixed thallium defects, the PCr/ATP ratio was significantly altered by exercise in patients with reversible thallium defects.
Yabe et al. [13]	1995	41 patients with CAD (severe LAD) 11 healthy controls	PCr content decreased significantly in patients with both reversible and fixed thallium defects ATP content decreased significantly in subjects with fixed thallium defects
Kalil-Filho et al. [14]	1997	8 healthy controls 29 patients with MI and successful reperfusion within 6 h of onset of chest pain	PCr/ATP ratios not significantly different between patients and controls or between early (4 days post MI) and late (39 days post MI) studies in patients.
Beer et al. [15]	2000	15 patients post MI (3 weeks post MI) 10 healthy controls	In viable myocardium, PCr/ATP did not have significant differences compared to healthy volunteers In akinetic myocardium, calculation of PCr/ATP ratio was not possible. However, a significant reduction of the ATP signal to noise ratio was observed
Buchthal et al. [16]	2000	35 women with normal coronary angiograms hospitalised for chest pain	Seven of the 35 women (20%) with chest pain and no angiographically significant stenosis had decreases in the PCr/ATP ratio during handgrip exercise that were more than 2 standard deviations below the mean value in the control subjects without chest pain
Beer et al. [17]	2004	8 patients with subacute inferior wall MI	PCr/ATP ratios of the inferior wall were significantly reduced compared to all other parts of the LV Transmural signal enhancement is associated with significant depression of PCr/ATP ratios
Bottomley et al. [18]	2009	15 patients with MI 15 healthy controls	PCr and ATP lower in patients with MI PCr/ATP normal in patients with MI CK flux halved in patients with MI
***Dilated Cardiomyopathy (DCM)***			
Schaefer et al. [6]	1990	14 healthy controls 11 patients with LVH, 6 patients with DCM	PCr/ATP ratio was not significantly different between patients and healthy individuals
Schaefer et al. [19]	1992	7 normal controls 8 patients with DCM (EF < 30%)	Subjects underwent dobutamine stress test In both normal and DCM groups, the PCr/ATP ratio showed no significant change between rest and peak stress.
de Roos et al. [20]	1992	9 healthy controls 9 patients with DCM 8 patients with HCM	PCr/ATP significantly lower in HCM patients relative to the control subjects but not in DCM patients
Neubauer et al. [21]	1995	14 healthy controls 23 patients with DCM	PCr/ATP ratio significantly reduced in DCM and correlated with the clinical severity of heart failure estimated from the NYHA class PCr/ATP correlated with left ventricular ejection fraction and left ventricular end-diastolic wall thickness
Neubauer et al. [22]	1997	39 patients with DCM (EF < 50%)	The lower the EF, the lower the PCr/ATP ratio The PCr/ATP ratio is a significant predictor of cardiovascular mortality
Beer et al. [23]	2002	10 healthy controls 10 patients with DCM	Both PCr and ATP concentrations correlated significantly with LV volumes, EF, and clinical status In DCM: PCr and ATP significantly reduced, the mild reduction of the PCr/ATP ratio underestimates true changes in heart failure due to simultaneous reductions of both PCr and ATP
Weiss et al. [24]	2005	16 healthy controls 16 patients with heart failure	Significant reduction in PCr and in CK flux and unchanged ATP in HF patients compared to controls
Hansch et al. [25]	2005	20 healthy volunteers 25 patients with DCM (15 with LVEF < 30% and severe symptoms, 10 with LVEF > 30% and moderate symptoms)	PCr/ATP ratio was significantly decreased in patients with moderate and severe DCM and showed a linear correlation with reduced LVEFs
Chida et al. [26]	2005	20 patients with global myocardial dysfunction (13 DCM, 3 HCM, 3 HHD, 1 AR)	PCr/ATP ratio was significantly reduced in patients with global myocardial dysfunction compared with healthy volunteers
Bottomley et al. [27]	2013	17 healthy subjects 58 patients with NICM	CK flux was significantly lower in HF patients Reduced myocardial CK flux was a significant predictor of all-cause mortality and HF outcomes, even after correction for NYHA class, LVEF, and race Risk of HF-related composite outcomes decreased by 32–39% for every 1 μmol g^−1^ s^−1^ increase in CK flux
Dass et al. [28]	2012	12 healthy 28 patients with HCM 18 patients with DCM	PCr/ATP ratio was significantly lower in both groups than in healthy subjects There was statistically significant negative correlation between T1 values and PCr/ATP ratios both for HCM and DCM groups
Dass et al. [29]	2015	12 healthy volunteers 14 patients with DCM	Resting PCr/ATP was reduced in DCM Oxygen supplementation did not change the PCr/ATP ratios in the DCM group
Schär et al. [30]	2015	12 healthy controls 17 patients with HF (LVEF < 40%)	CK flux was significantly reduced in HF patients
Stoll et al. [31]	2016	10 healthy controls 25 patients with DCM	Patients with dilated cardiomyopathy had a significantly lower PCr/ATP than did healthy control subjects at 7 T
Gabr et al. [32]	2018	14 healthy volunteers 27 patients with mild-to-moderate HF (EF ≤ 45%)	PCr and ATP and the CK rate-constant *k_f_* were all modestly reduced in HF patients by 13–19%. CK flux was significantly reduced by 32% In addition, cardiac CK flux correlated with the resting peak and average mechanical power and with mechanical efficiency
Rayner et al. [33]	2022	26 healthy controls 16 patients with DCM 27 patients with DCM and obesity	PCr/ATP ratio and CK flux was significantly lower in DCM compared with controls CK flux was two-fold higher in obese DCM During increased workload, CK flux increased in controls, remained unchanged in DCM normal and fell in obese DCM Following weight loss PCr/ATP did not fall during increased workload, while CK flux increased Myocardial PCr/ATP was reduced in DCM Weight loss intervention prevented fall in PCr/ATP ratio and increased median CK flux during dobutamine stress test.
***Hypertrophic Cardiomyopathy (HCM)***			
Sakuma et al. [34]	1993	6 healthy controls 19 patients with HCM	PCr/ATP ratio significantly lower in HCM patients
Sieverding et al. [35]	1997	16 healthy controls 13 patients with HCM	No differences in PCr/ATP between the groups PCr/P_i_ ratio significantly lower in HCM group
Jung et al. [36]	1998	11 healthy controls 14 patients with HCM	Patients with HCM have significant lower PCr/ATP ratio
Okada et al. [37]	1998	30 healthy control subjects 10 patients with HCM	In the healthy volunteers: PCr and ATP concentrations decreased significantly with age In HCM: reduced ATP and PCr concentrations
Grilley et al. [38]	2003	24 healthy controls 31 patients with HCM mutation positive (beta-myosin heavy chain, cardiac troponin T, or myosin-binding protein C)	PCr/ATP was significantly reduced in the HCM subjects relative to controls (by 30%) The reduction was of a similar magnitude in all three disease-gene groups
Shivu et al. [39]	2008	37 healthy volunteers 26 patients with hypertrophic cardiomyopathy (HCM)	The PCr/ATP ratio was significantly reduced in HCM patients compared to controls
Esposito et al. [40]	2009	19 healthy controls 19 patients with HCM	PCr/ATP ratio was lower in HCM patients than in control subjects. LE% and PCr/ATP-ratio were inversely related LE% was the stronger predictor of PCr/ATP ratio by multivariate analysis.
Dass et al. [28]	2012	12 healthy controls 28 patients with HCM 18 patients with DCM	PCr/ATP ratio was significantly lower in both groups than in healthy subjects There was statistically significant negative correlation between T1 values and PCr/ATP ratios both for HCM and DCM groups
Abraham et al. [41]	2013	17 healthy controls 9 patients with HCM with point mutation (Arg403Gln) in the cardiac β-myosin heavy-chain (MHC) gene	PCr lower in HCM patients PCr/ATP ratio was not statistically lower but there was a trend CK flux was significantly reduced
Dass et al. [42]	2015	20 healthy volunteers 35 patients with HCM	Resting PCr/ATP was significantly reduced in HCM During exercise, there was a further reduction in PCr/ATP in HCM but not in normal
Valkovic et al. [43]	2019	10 healthy controls 3 patients with HCM	PCr/ATP lower in HCM patients Pi/PCr and Pi/ATP were higher in HCM patients
***Heart Failure with Preserved Ejection Fraction (HFpEF), Left ventricular hypertrophy, Hypertensive Heart Disease (HHD), Diabetic Cardiomyopathy, Obesity***			
Okada et al. [37]	1998	30 healthy control subjects 8 patients with HHD	In the healthy volunteers: PCr and ATP concentrations decreased significantly with age In HHD: PCr and ATP concentrations were similar to the healthy group
Lamb et al. [44]	1999	11 patients with HTN 13 healthy controls	PCr/ATP was significantly lower in HTN group both at rest and during stress PCr/ATP acquired at rest was correlated with LV diastolic function indexes determined at rest
Beer et al. [23]	2002	10 healthy controls 10 patients with HHD	Both PCr and ATP concentrations correlated significantly with LV volumes, EF, and clinical status In HHD: PCr, ATP and PCr/ATP were unchanged
Heyne et al. [45]	2006	20 healthy controls 36 patients with HHD (11 systolic dysfunction)	PCr/ATP ratio was significantly reduced in HHD PCr/ATP ratio was significantly lower in patients with systolic dysfunction compared to those without PCr/ATP ratio was correlated linearly with LVEF
Smith et al. [46]	2006	14 healthy controls 20 patients with LVH (10 of which LVH + CHF)	PCr concentrations lower in LVH patients, ATP similar to healthy volunteers PCr/ATP significantly lower in LVH group CK flux lower in LVH with the reduction more pronounced in the LVH + CHF group
Phan et al. [47]	2009	20 controls 37 patients with HFpEF	PCr/ATP ratio in patients with HFpEF was significantly reduced compared with healthy control subjects
Levelt et al. [48]	2016	20 healthy controls 46 patients with DMII	PCr/ATP ratio was significantly lower in patients with DMII
Rayner et al. [49]	2020	80 volunteers (35 controls, 45 obese)	At rest, obesity was associated with reduced PCr/ATP During increased workload, CK flux failed to increase in obese Weight loss was associated with an increase in PCr/ATP and was associated with increase in CK flux during increased workload
Burrage et al. [50]	2021	11 healthy controls 32 patients with diastolic dysfunction and HFpEF (9 with DMII, 14 with HFpEF, 9 with severe diastolic dysfunction attributable to cardiac amyloidosis)	PCr/ATP ratio was significantly reduced in all groups compared with controls PCr/ATP ratio correlated with log NT-proBNP echocardiographic E/e′ ratio, NYHA class and exercise induced pulmonary congestion
Thirunavukarasu et al. [51]	2021	Empagliflozin on DMII 18 DMII 12 weeks	PCr/ATP reduced in patients with DMII empagliflozin led to significant improvements in myocardial energetics
Rayner et al. [33]	2022	26 healthy controls 16 patients with DCM 27 patients with DCM and obesity	PCr/ATP ratio and CK flux was significantly lower in DCM compared with controls CK flux was two-fold higher in obese DCM During increased workload, CK flux increased in controls, remained unchanged in DCM normal and fell in obese DCM Following weight loss PCr/ATP did not fall during increased workload, while CK flux increased Myocardial PCr/ATP was reduced in DCM Weight loss intervention prevented fall in PCr/ATP ratio and increased median CK flux during dobutamine stress test.
***Valvular Heart Disease***			
Conway et al. [52]	1991	13 healthy controls 8 patients with AS, 8 patients with AR (out of which 6 had HF symptoms, for which they were on medical treatment)	PCr/ATP significantly lower in the symptomatic patients with aortic valve disease compared to controls and to those without symptoms
Neubauer et al. [53]	1997	13 patients with AS 9 patients with AR	For all patients, PCr/ATP ratio was significantly reduced with NYHA class III but not with I or II PCr/ATP was significantly reduced in AS patients while in AR the ratio only showed a trend for reduction In AS, PCr/ATP ratio was significantly reduced only when LV end-diastolic pressures were > 15 mm Hg or when LV diastolic wall stress was > 20 kdyne cm^−2^
Conway et al. [54]	1998	13 healthy controls 22 patients with chronic MR	PCr/ATP ratio was significantly lower in severe disease, symptomatic and those on anti-HF therapy compared to control and asymptomatic patients. PCr/ATP correlates with echo derived dimensional indexes of left ventricular dilatation (The greater the LV dilatation, the lower the ratio)
Beer et al. [23]	2002	10 healthy controls 10 patients with aortic stenosis (AS)	Both PCr and ATP concentrations correlated significantly with LV volumes, EF, and clinical status In AS: PCr significantly reduced, ATP and PCr/ATP showed a trend for reduction
Mannacio et al. [55]	2012	30 patients post AVR (Medtronic Mosaic bioprosthesis) 15 with PPM, 15 without PPM	PCr/ATP improved significantly post AVR in both groups Significant correlation between PCr/ATP and left ventricular diastolic function No significant correlation between the PCr/ATP ratio and left ventricular mass
Mahmod et al. [56]	2014	15 healthy controls 28 patients with severe AS	PCr/ATP ratio was significantly lower than in healthy subjects There is significant (inverse) correlation between PCr/ATP ratio and circumferential strain PCr/ATP ratio significantly improved 8 months post AVR
Peterzan et al. [57]	2020	30 healthy 13 patients with moderate AS 37 patients with severe AS and EF ≥ 55% 15 patients with severe AS and EF < 55% 7 pressure-loaded heart biopsies	Myocardial PCr/ATP ratios in all AS groups, including severe AS and reduced EF, were lower than that recorded in the nonhypertrophied heart PCr/ATP ratio and CK flux significantly reduced in moderate and severe AS with normal EF compared with normal controls (pressure loading was associated with altered myocardial energetics)
***Heart transplant***			
Evanochko et al. [58]	2002	11 healthy controls 25 heart transplant patients	10 patients positive ^31^P MRS stress test for ischaemia with a significant change (reduction) in PCr/ATP ratio
Caus et al. [59]	2006	9 healthy controls 8 patients with CAV (cardiac allograft vasculopathy) 18 patients without CAV	PCr/ATP ratio was significantly reduced in CAV PCr/ATP value of 1.59 was the optimal cut-off value to predict CAV (specificity and sensitivity of 100% and 72%, respectively).
***Pharmaceutical Interventions and Research Trials***			
Lee et al. [60]	2005	Double blind RCT in patients with CHF 28 patients received placebo 28 patients received perhexiline (antianginal drug that augments glucose metabolism by blocking muscle mitochondrial free fatty acid uptake and thereby increasing metabolic efficiency)	Treatment with perhexiline normalised skeletal muscle phosphocreatine recovery after exercise Perhexiline led to significant improvements in Vo_2_max quality of life and left ventricular ejection fraction increased resting and peak dobutamine stress regional myocardial function
Fragasso et al. [61]	2006	12 patients with HF randomized in a double-blind, cross-over study to Placebo or trimetazidine (20 mg t.i.d.) for two periods of 90 days	PCr/ATP ratio significantly increased with trimetazidine (along with improvement of EF and NYHA class
Hirsch et al. [62]	2012	Double blind randomised placebo-control study 16 patients with NICM randomised to allopurinol (with a ratio 4:1)	Allopurinol infusion increased mean cardiac PCr/ATP and PCr concentration by ~11% and mean CK flux by 39%.
Spoladore et al. [63]	2013	10 HF patients had beta blocker (bisoprolol or carvedilol)	PCr/ATP ratio increased by 33% after 3 months of beta blocker
Beadle et al. [64]	2015	Double blind randomised placebo-control study 50 patients with NICM (symptomatic and LVEF < 40%) randomised to placebo (25) and perhexiline (25)	Significant difference in mean PCr/ATP ratio between patients in lower and higher NYHA functional classes (baseline) At follow-up, the myocardial PCr/ATP ratio increased by 30% versus baseline in the perhexiline group and did not change in the placebo group
Thirunavukarasu et al. [51]	2021	Empagliflozin for 18 patients with DMII for 12 weeks	Empagliflozin led to significant improvements in myocardial energetics
***Additional Studies***			
Leme et al. [65]	2013	28 patients with Chagas disease 8 healthy controls	PCr/ATP reduced significantly at rest (lowest in those with systolic dysfunction) Regardless of left ventricular function, Chagas patients exhibit an exercise-induced decline in cardiac high-energy phosphates (PCr/ATP) consistent with myocardial ischemia
Solaiyappan et al. [66]	2019	109 patients with NICM (38 DCM, 32 HCM) 15 patients with CAD 45 healthy controls	Neural networks employing 31P-MRS measures (ATP, PCr, the first order CK reaction rate kf, CK flux) had an accuracy of 84% in discriminating healthy, HF and non-HF cardiac disease

AS aortic stenosis; AR aortic regurgitation; AVR aortic valve replacement; CAD Coronary Artery Disease; CAV Cardiac allograft vasculopathy; CHF Congestive Heart Failure; DMII Diabetes Mellitus Type II; EF ejection fraction; HCM Hypertrophic cardiomyopathy; HHD Hypertensive Heart Disease; ICM Ischaemic Cardiomyopathy; LAD (Left Anterior Descending Artery); LVH (Left Ventricular Hypertrophy); MI (Myocardial Infarction); MR (Mitral Regurgitation); NICM (Non-ischaemic Cardiomyopathy); PPM (Patient-Prosthesis Mismatch)

## Data Availability

All data are available from the corresponding author on request.
